# The evolutionary process of invasion in the fall armyworm (*Spodoptera frugiperda*)

**DOI:** 10.1038/s41598-022-25529-z

**Published:** 2022-12-06

**Authors:** Sudeeptha Yainna, Wee Tek Tay, Karine Durand, Estelle Fiteni, Frédérique Hilliou, Fabrice Legeai, Anne-Laure Clamens, Sylvie Gimenez, R. Asokan, C. M. Kalleshwaraswamy, Sharanabasappa S. Deshmukh, Robert L. Meagher, Carlos A. Blanco, Pierre Silvie, Thierry Brévault, Anicet Dassou, Gael J. Kergoat, Thomas Walsh, Karl Gordon, Nicolas Nègre, Emmanuelle d’Alençon, Kiwoong Nam

**Affiliations:** 1 DGIMI, Univ Montpellier, INRAE, Montpellier, France; 2 CIRAD, UPR AIDA, Montpellier, France; 3 Black Mountain Laboratories, CSIRO, Canberra, Australia; 4 INRAE, Institut Sophia Agrobiotech, Université Côte D’Azur, CNRS, Sophia Antipolis, France; 5 INRAE, UMR-IGEPP, BioInformatics Platform for Agroecosystems Arthropods, Campus Beaulieu, 35042 Rennes, France; 6 INRIA, IRISA, GenOuest Core Facility, Campus de Beaulieu, Rennes, France; 7 CBGP, INRAE, CIRAD, IRD, Institut Agro, Univ Montpellier, Montpellier, France; 8 Division of Biotechnology, ICAR - Indian Institute of Horticultural Research, Bengaluru, India; 9 Department of Entomology, College of Agriculture, University of Agricultural and Horticultural Sciences, Shivamogga, India; 10 United States Department of Agriculture, Agricultural Research Service, Gainesville, FL USA; 11 United States Department of Agriculture, Animal and Plant Health Inspection Service, Maryland, USA; 12 AIDA, Univ Montpellier, CIRAD, Montpellier, France; 13 PHIM, Univ Montpellier, IRD, CIRAD, INRAE, Institut Agro, Montpellier, France; 14 ENSBBA, UNSTIM, Dassa, Benin

**Keywords:** Invasive species, Agroecology, Evolutionary genetics, Population genetics

## Abstract

The fall armyworm (FAW; S*podoptera frugiperda*) is one of the major agricultural pest insects. FAW is native to the Americas, and its invasion was first reported in West Africa in 2016. Then it quickly spread through Africa, Asia, and Oceania, becoming one of the main threats to corn production. We analyzed whole genome sequences of 177 FAW individuals from 12 locations on four continents to infer evolutionary processes of invasion. Principal component analysis from the TPI gene and whole genome sequences shows that invasive FAW populations originated from the corn strain. Ancestry coefficient and phylogenetic analyses from the nuclear genome indicate that invasive populations are derived from a single ancestry, distinct from native populations, while the mitochondrial phylogenetic tree supports the hypothesis of multiple introductions. Adaptive evolution specific to invasive populations was observed in detoxification, chemosensory, and digestion genes. We concluded that extant invasive FAW populations originated from the corn strain with potential contributions of adaptive evolution.

## Introduction

Biological invasion by pest species is one of the main causes of economic losses in agriculture^1–4^. As human trade is constantly increasing, the number of reported cases of invasive species has consequently surged up^5,6^, particularly in insect species^7^. Indeed, insects comprise 88.24% of reported invertebrate invasive cases in the Global Invasive Species Database^8^ (accessed on 27th July 2021). Consequently, invasive pest insects cause serious losses in major crop production^9^, especially in sub-Saharan African countries, where the national economy is heavily dependent on agriculture, as up to 35% of national gross domestic product (GDP) is lost due to invasive pests^10^.

The process of an invasion typically involves three key steps^11^. The first step is the introduction of an alien species outside its native range. Invasive species are often introduced through anthropogenic transport, such as trade, urbanization, irrigation, and roads and railways^12^. The second step is the establishment of stable populations, thanks to a higher intrinsic growth rate^13^ or a higher competitive ability^14^ than native species. The third step is range expansion from the established population. Adaptive evolution may contribute to all of these steps by (i) fixation of beneficial de novo mutations or standing genetic variations, (ii) hybridization between introduced and native species, or (iii) genome doubling (reviewed in^11^). A time gap between introduction and range expansion has been often observed, and this gap period was coined the ‘lag phase’^15^. Since the eradication of an introduced pest species is only realistic during its lag phase, it is important to identify alien species before they become invasive for pest management.

The fall armyworm, *Spodoptera frugiperda* (J.E. Smith) (FAW, Lepidoptera: Noctuidae: Noctuinae), is one of the most infamous insect pests due to its severe impact on major crops including corn, cotton, rice, sorghum, and soybean^16,17^. The FAW exhibits high dispersal ability combined with a marked migratory behavior^18^, the ability to rapidly develop resistance against synthetic insecticides^19^ and *Bacillus thuringiensis* proteins^20–22^, alongside the potential for occasional outbreaks. The FAW consists of two host plant strains with differentiated ranges of host plants, the corn strain (sfC) and the rice strain (sfR) (named after their preferred host plants). These two strains are observed in sympatry throughout their native range^23–25^. Since sfC and sfR are morphologically indistinguishable, the two strains can only be identified with molecular markers^26,27^. The nuclear triosephosphate isomerase (TPI) gene on Z chromosome^28^ and the mitochondrial cytochrome *c* oxidase subunit 1 (COX1) gene^27,29^ are commonly used for this purpose.

The FAW is native to the Americas, and its invasion of the Old World was first reported in West Africa in 2016^30^. In the years following its first report, FAWs were detected throughout sub-Saharan Africa, followed by their widespread detection in India, South East Asia, East Asia, Egypt, and Oceania (https://www.cabi.org/isc/fallarmyworm). Invasive FAW larvae cause significant economic losses, especially on corn with a production yield loss of 21–53% in Africa^31^, where corn is one of the most important staple crops (more than 30% of total caloric intake comes from corn^32^). Chemical insecticides are commonly used to control invasive FAW populations, but field-evolved insecticide resistance against chemical insecticides has been reported from Chinese populations^33,34^.

Since the global invasion of FAWs is a pressing issue due to the severe economic impacts, there is an urgent need to obtain information on the population structure of invasive and native FAW populations in the context of geography and host plants. Gui et al.^35^ reported that the Chinese populations most likely originated from African populations using population genomics analyses. Schlum et al.^36^ also performed population genomics analyses of one invasive (Kenya) and four native (Argentina, Brazil, Puerto Rico, and mainland USA) populations, but they observed no clear pattern of population structure. Since these studies are based on the FAW samples collected from corn or sorghum, which are presumably the preferred plants of sfC^25^, the genetic relatedness among native sfC, native sfR and invasive FAW populations has yet to be determined in these studies.

Even though invasive FAW populations are found almost exclusively in sfC-preferred plants^37–39^, such as corns and sorghums, several genetics studies argued that invasive FAWs are hybrids between sfC and sfR. Marker-based studies showed that most invasive FAWs are sfC according to the mitochondrial COX1 genes and sfR according to the TPI genes, arguing that invasive FAWs were generated through interstrain hybridization^40,41^. But it is still unclear whether the same pattern can be supported if whole genome sequences are analyzed. Zhang et al*.* reported through genomics analysis that Chinese FAW populations are hybrids between sfC and sfR^33^. However, since they included only one sfC sample and one sfR sample from native populations, it is still unclear whether the same pattern will be observed when population data from native population are included. In short, the argument that invasive FAWs are hybrids between sfC and sfR needs to be revisited using population genomics data. If invasive FAWs are indeed hybrids, it is not easy to explain why they are found almost exclusively in sfC-preferred host plants.

In this study, we aim at inferring the population structure of FAW populations, including native sfC, sfR, and invasive populations using whole genome sequences from samples collected globally from North and South America (native range), East and West Africa, and South and East Asia (invasive ranges). Native FAW samples were collected from both corn (sfC preferred plants), as well as grass or rice (sfR preferred plants). We also identified loci with signatures of adaptive evolution that may contribute to the FAW invasive success.

## Results

### Invasive origins

**Figure 1:**
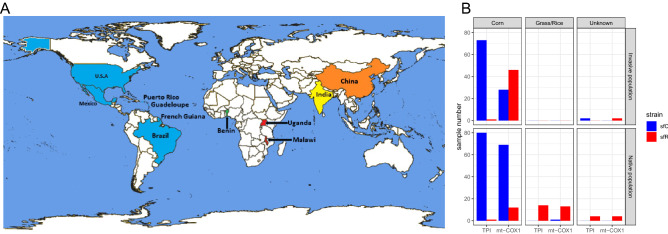
**Samples used in this study** (**A**) Map indicating the countries from which samples of *Spodoptera frugiperda* were sequenced. The blue color indicates the native countries of *S. frugiperda*. The green, red, yellow, and orange colors indicate that the invasion was reported in 2016, 2017, 2018, and 2019 respectively. The map was generated using MapChart^42^. (**B**) The numbers of sfC and sfR samples according to the host plants using different diagnostic markers (TPI or mitochondrial COX1).

The total number of individuals used in this study was 177 (99 from native populations and 78 from invasive populations) from 12 geographic populations (Fig. 1A and Table 1), making this dataset the largest and most comprehensive to date. The identified strain had an almost perfect correlation with host plants (Fig. 1B) according to the TPI marker. When the mitochondrial COX1 marker was used, almost all samples from rice or grasses were sfR, but sfR was also often observed from corn plants.

**Table 1:** Information of resequencing data used in the study.

Country	District/village	The number of samples	Source
India	Karnataka	14	^43^
China	Kunming	2	^44^
Mexico	Texcoco	26	Current study
French Guiana		3	Current study
Guadeloupe	Petit-Bourg, Port-Louis	4	Current study
Puerto Rico	Santa Isabel	15	^45^
USA	Mississippi—Stoneville	17	^46,47^
	Florida—Citra and Jacksonville	24	^48^
Brazil	State of Goiás	10	^49,50^
Malawi	Blantyre, Chiradzulu, Machinga, Mulanje, Thyolo, Zomba, Salima, Karonga, Mzimba, Nkhata Bay	16	^49,50^
Uganda	Amolatar, Katakwi, Kumi, Ngora, Pallisa, Soroti, Mbarara, Wakiso	7	^49,50^
Benin	Wagou, Gando	10	Current study

**Figure 2:**
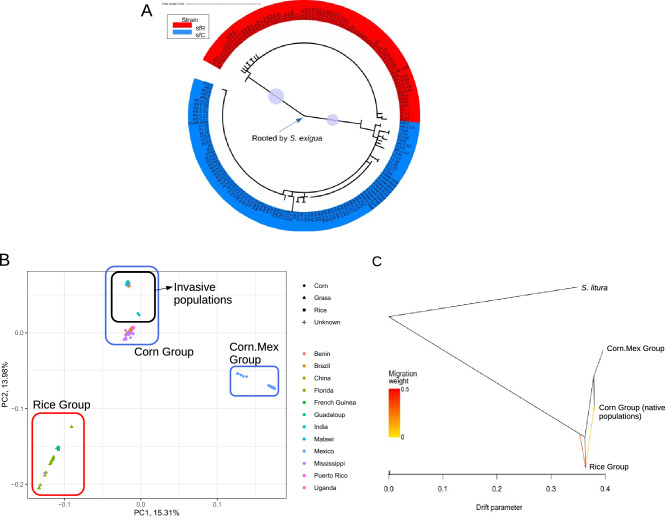
**Invasive origins inferred from mitochondrial or nuclear sequences** (**A**) Maximum-likelihood phylogenetic tree was reconstructed from the full-length mitochondrial COX1 gene (1536 bp). The red and blue clades indicate sfR and sfC, respectively. The circles on the branches show bootstrap support values higher than 90%. A branch leading to *Spodoptera exigua* was deleted while the position was indicated for a visualization purpose. (**B**) The result of the principal component analysis showed that FAW populations are composed of Rice, Corn, and Corn.Mex groups and that invasive populations belong to the Corn groups. (**C**) TreeMIX analysis shows that the Corn group was derived from the ancestry of the Corn.Mex and the Corn groups. The arrows indicate the direction of gene flow, which was detected from the Rice group to the Corn group. The color gradient of the arrows indicate the migration weight.

The mitochondrial COX1 phylogenetic tree recovered two clades corresponding to sfC and sfR mitochondrial strains with high bootstrap confidence scores (bootstrap support value of 97.3%) (Fig. 2A). The existence of two mitochondrial strains in invasive populations suggests a possibility of multiple introductions from the Americas to the Old World**,** as shown by Tay et al.^49^.

A principal component analysis was performed using 27,117,672 nuclear SNVs to infer FAW population structure. The first principal component explained 15.31% of the variance in allele frequency (Fig. S1A), of which the eigenvectors displayed three groups of individuals (Figs. 2B, S1B). The first group (referred to as ‘Rice group’ in this paper) consisted of samples collected from rice or grasses in the Caribbean (including Florida, Guadeloupe) and French Guiana, but also an individual from corn in the Mississippi. The second group (referred to as ‘Corn.Mex group’) consisted of samples from corn in Mexico only. The third group was found between the first and second groups along the first principal component. This group was composed of samples from corns only (referred to as ‘Corn group’).

The first principal component in Fig. 2B did not separate invasive populations (Benin, Malawi, Uganda, India, and China) from any native populations. But invasive populations were separated from the native populations in the Corn group along the second principal component, which explained 13.98% of the variance in allele frequency. F_ST_ statistics show significant genetic differentiation between the native and invasive populations in the Corn group (F_ST_ = 0.0432; p < 0.01). This result shows that genetic variations can be explained primarily by three groupings (e.g., Rice group, Corn.Mex group, and Corn group) and that the invasive populations were derived from the native population(s) in the Corn group.

A whole nuclear genome phylogenetic tree reconstructed from native FAWs showed that ancestral FAWs split between the Rice group and (the Corn.Mex + the Corn groups) in the area of origin, and a unidirectional gene flow from Rice group to Corn group was observed when the number of migration edges was six (Fig. 2C). This phylogenetic relationship was consistently observed when different numbers of migration edges were assumed (Fig. S2). This result shows that the Corn group was derived from the ancestry of the Corn and Corn.Mex groups.

### A single ancestry of invasive FAWs

**Figure 3:**
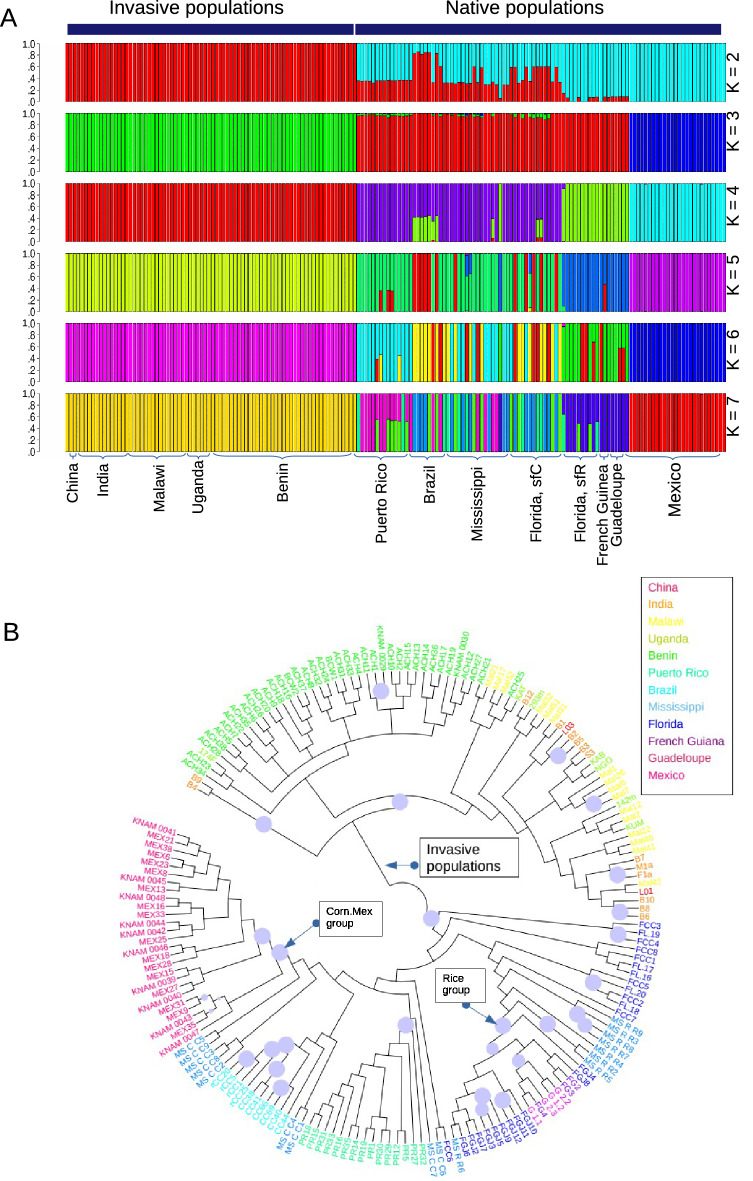
**A single origin of invasive populations** (**A**) Ancestry coefficient analysis from SNVs showed homogeneous genomic sequences of invasive populations. (**B**) A nuclear BIO-NJ phylogenetic tree was reconstructed from whole nuclear genome sequences. The circles on the branches show bootstrap support values higher than 90%. The Corn.Mex group and the Rice group are indicated to the corresponding nodes. The remaining individuals belong to the Corn Group. Invasive individuals were also indicated to the corresponding node. The phylogenetic tree was visualized using iTOL v4^52^.

We performed the ancestry coefficient analysis^51^ to infer the ancestry of each individual. Invasive populations appeared to share a single common ancestor, distinct from all native populations, in a range of K values (Figs. 3A and S3). The single ancestry of invasive populations was further tested by reconstructing a BIO-NJ phylogenetic tree inferred from all individuals. If all the invasive individuals from populations originated from a single ancestry, these individuals will show monophyly with the assumption that invasive populations originated from native populations. The resulting tree exhibited a clade composed of invasive individuals with 100% of the bootstrapping support, and this clade does not contain any individuals from native populations (Fig. 3B). This result shows the monophyly of invasive populations.

If invasive populations experienced genetic admixture between two sources with different mitochondrial strains (Fig. 2A), then invasive populations are expected to have lower D_XY_ between the mitochondrial strains than native populations due to the gene flow^53^. F_ST_ statistics showed nuclear genomic differentiation between the mitochondrial strains both in the invasive populations (F_ST_ = 0.00281; p < 0.01) and in the native populations (F_ST_ = 0.0124; p < 0.01) (Fig. S4A). Invasive populations had lower D_XY_ than native populations across whole genomes (Fig. S4B), suggesting that invasive populations experienced an increased rate of genetic exchanges between mitochondrial strains.

### Adaptively evolved genes in the invasive population

**Figure 4:**
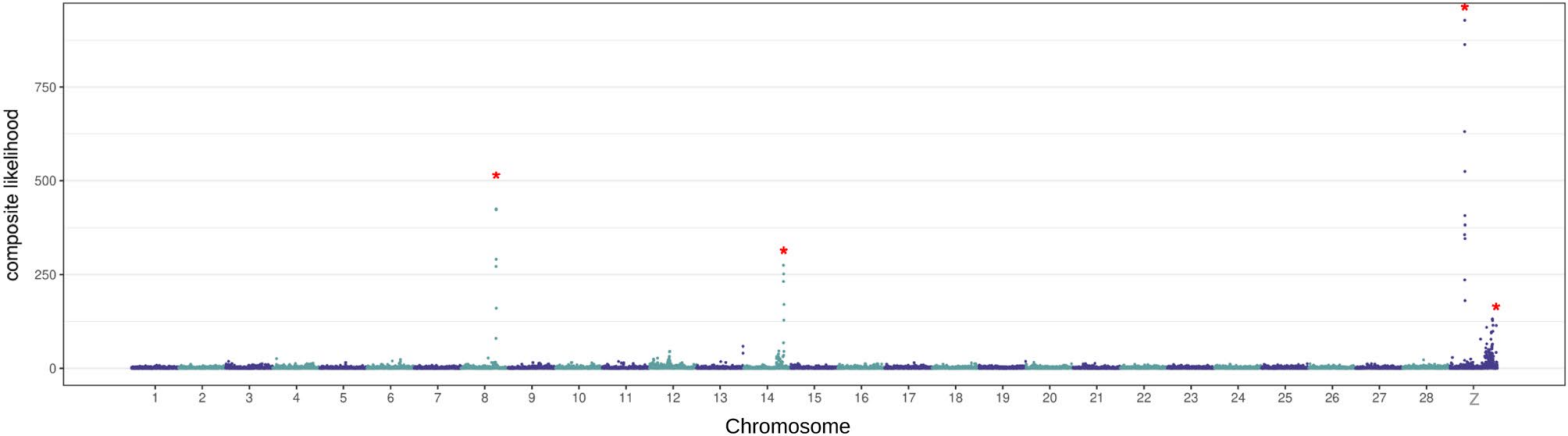
**Loci under adaptive evolution** The composite likelihood of being targeted by selective sweeps in invasive populations. The x-axis represents the coordinates of grids in the reference genomes, and the y-axis is the composite likelihood of each grid. The red asterisks indicate potential targets of invasive population-specific selective sweeps.

We tested if adaptive evolution specific to invasive populations is supported using the composite likelihood of selective sweeps^54^. The median value of the composite likelihood was 0.4350. A locus was considered to be targeted by selective sweep if the composite likelihood was higher than 100, a threshold that was arbitrarily chosen. We were able to pinpoint the apparent outlier of the composite likelihood through eyeballing as well. In total, 26 grids had a composite likelihood higher than 100. These grids represented 0.090% of the total 29,000, meaning that a very stringent criterion was applied here to infer loci under selective sweeps. Neighboring grids constituted seven loci on three chromosomes. We considered that these loci are potential targets of selective sweeps (Fig. 4). As the high composite likelihood of these loci could be generated by selective sweeps not specific to invasive populations or by background selection^55^, we also calculated the composite likelihood from native individuals from the Corn group. Four out of the seven loci did not exhibit outliers of the composite likelihood in the native population in the Corn group (Fig. S5). We, therefore, considered that these four loci were potentially targeted by selective sweeps specific to invasive populations. These four loci contained 36 predicted protein-coding genes (Table S1), including 12 genes with unknown functions. We carefully underwent a manual curation of these genes by NCBI blasting to determine their functions. The locus on chromosome 14 encompasses CYP9A, which belongs to the Cytochrome P450 gene family. A locus on the Z chromosome includes a carboxylesterase gene, an ABC transporter homolog to mdr49, a kunitz-type serine protease inhibitor gene, odorant receptor 13, and the *clk* gene.

## Discussion

Invasive FAW insects have been one of the main threats against corn production across the Old World. In native areas, FAW is composed of two sympatric strains with differentiated host plants, such that sfC prefers corns and sfR prefers rice and grass^23^. Population structure among invasive FAWs, native sfC, and native sfR remained elusive. In this study, we inferred the population structure of FAW using whole genome sequences from 177 individuals collected from 12 geographic locations in native and invasive areas. Principal component analysis from the whole nuclear genome demonstrates that invasive FAWs originated from the corn-eating group (i.e., the Corn group) (Fig. 2B). The phylogenetic tree in Fig. 2C shows that the Corn group was derived from the ancestry of the Corn + Corn.Mex groups. We argue that the Corn group in Fig. 2B should be considered to be sfC, even though a proportion of this group was classified as sfR according to the mitochondrial COX1 marker because these samples were sampled only from corns. We reported that the TPI marker can be reliably used to identify host plant strains (sfC or sfR) whereas the COX1 marker should be used to identify two phylogenetic entities within sfC^48^. In this study, according to the TPI marker, almost all invasive FAWs were classified as sfC (Fig. 1B), in line with the conclusion that invasive FAWs should be considered to be sfC. The reason why invasive FAWs are found almost exclusively in sfC-preferred host plants is probably due to the fact that they are sfC. Therefore, we argue that invasive FAWs should not be considered to be hybrids anymore^33,40,41^.

Interestingly, the analyzed invasive FAWs appear to originate from a single population (Fig. 3) even though the mitochondrial phylogenetic tree shows that invasive FAWs are introduced from at least two genetic entities (e.g., sfC and sfR according to the mitochondrial COX1 marker) (Fig. 2A), as previously shown by Tay et al.^49^. Invasive populations appear to have experienced genetic admixture between these genetic entities with different mitochondrial markers (Fig. S4). We postulate that if FAWs were introduced from multiple origins a long time before 2016, when FAW invasion was first reported in West Africa^30^, the introduced FAWs might have had enough time to experience genetic admixtures. According to this hypothesis, introduced FAWs had genetically admixed nuclear genomes through recombination while non-recombining mitochondrial genomes were still distinctly separated. During this time of the lag phase, the introduced FAWs might have been undetected, possibly due to small population sizes before experiencing a population explosion in 2016. The presence of lag phases has been reported mostly in plants^56^, but also in insects^57^, birds^58^, and fishes^59^. In the FAWs, the existence of the lag phase should be formally tested in future studies.

### Adaptive evolution contributed to invasive success

The identified genes under invasive population-specific selective sweeps (Fig. 4) suggest the possibility that insecticide resistance has contributed to the FAW invasive success. For example, the CYP9A gene on chromosome 14 may play an important role in host plant adaptation or insecticide resistance. CYP9A belongs to the cytochrome P450 gene family, a key player in the detoxification of xenobiotics^45,60^. CYP9A genes are overexpressed by plant allelochemicals and pesticides in FAW^61^, and CYP9A gene amplification has been observed in Puerto Rico^45^, where extensive field-evolved resistance against various insecticides has been reported in FAW^19^. A selectively targeted locus on the Z chromosome includes the carboxylesterase gene, which may be also involved in insecticide resistance^62^. Therefore, insecticide resistance could be one of the key selective pressures for invasion success. In our previous study, we observed that invasive FAW populations have higher gene copy numbers of CYP genes than native populations and that invasive populations have higher allele frequencies causing insecticide resistance^63^, consistent with the argument that insecticide resistance may have contributed to the invasive success. Indeed, invasive FAW populations have shown to have resistance against insecticides^33,34^.

The locus on the Z chromosome also includes an ABC transporter homolog to mdr49, which protects organisms from cytotoxic compounds in *Drosophila melanogaster*^64^. This locus also includes a Kunitz-type serine protease inhibitor gene, which plays a role in plant digestion^65^, and a gene encoding odorant receptor 13, which may be important for the selection of foraging or oviposition sites^66^. Since detoxification genes, digestion genes, and chemosensory genes are the key genetic elements determining the range of host plants in the FAW^46^, selective sweeps on the locus including these genes may suggest the possibility that invasive FAWs experienced adaptation to host plants.

Another locus on the Z chromosome under selective sweeps includes *clk*, a key circadian clock gene^67^. Native populations have different mating times between sfC and sfR^68,69^, while invasive African populations of FAWs do not exhibit such different mating times while having earlier mating times than American populations by three hours^70^. The genetic differentiation of *clk* from native populations could promote hybridization between sfC and sfR through changes in mating time in African populations. Further genomics and behavior studies are required to test the association between genotypes at the *clk* gene and mating behavior.

### Process of FAW invasion

**Figure 5:**
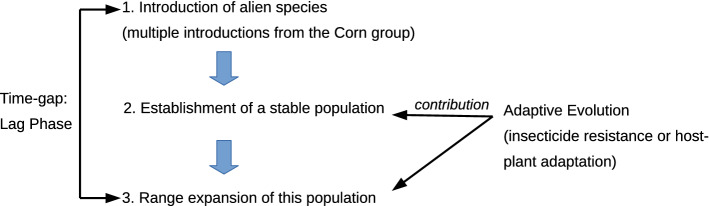
**Models of the invasive process in FAW** First, FAWs were introduced from the Corn group between sfC and sfR, possibly multiple times. Second, a stable population was established in a location in the Old World. This population experienced growth in size, and FAW invasion was first reported. Third, range expansion occurred from this population. The first step occurred before 2016, implying the existence of a lag phase. Adaptive evolution contributed to the second and third steps, potentially through insecticide resistance or host plant adaptation.

Taken together, we propose the following evolutionary invasive scenario (summarized in Fig. 5). First, FAW has been introduced in the Old World multiple times. The introduced FAWs experienced a lag phase^15^ and genetically admixed FAWs were generated. Second, a stable population at one location in the Old World was established, and this population experienced explosive growth in population size. At this time, the FAW invasion was first reported^30^. Third, invasive FAWs exhibited very rapid range expansion across sub-Saharan Africa, Asia, Oceania, and Egypt. We also argue that this proposed evolutionary history can be promoted by adaptive evolution, especially in the second and third stages.

The observed genetic differentiation between the analyzed invasive and native populations in the Corn group could be caused by multiple evolutionary forces. First, newly generated mutations during a lag phase might have contributed to differentiation. Second, multiple introductions of FAWs themselves may generate the observed genetic differentiation since genetically admixed individuals have differentiated genotypes from each of the origins. Third, mild positive selection targeting many genes may have accelerated the genomic differentiation between invasive and native populations through synergistic effects among linkage disequilibrium^47,71^. Since these explanations are not mutually exclusive, it is possible that these three evolutionary forces simultaneously increased genetic differentiation between invasive and native populations.

### Potential issues of sampling bias

Since our sampling does not cover the entire range of FAW (especially in its native range in the Americas), the patterns observed in this study could be affected by temporal or geographical sampling bias, even though the analyzed FAWs were collected from a wide range of geographic locations in four continents. Since geography does not have a major effect on the population structure within the Corn group (Fig. 3A), we do not believe that an unknown geographic population in the Corn group has different genomic sequences from all samples analyzed in the native Corn group while being highly similar to the invasive Corn groups. However, it may be possible to pinpoint the exact invasive origins in native areas if additional geographic populations are included in future studies.

## Conclusion

In this study, we showed that invasive FAWs originated from sfC in the native area. The invasive FAWs appear to be derived from a single origin despite multiple introductions, suggesting the possibility of genetic admixture during a lag phase. We also observed footprints of adaptive evolution specific to invasive populations. Adaptive evolution specific to the identified invasive populations may have caused insecticide resistance or host plant adaptation, possibly contributing to invasive success. We argue that comparative genomics and functional studies on host plant adaptation and insecticide resistance are urgently needed to identify the main evolutionary selective forces responsible for invasive success, as this information may be useful to better control FAW in the Old World.

## Materials and methods

### Sampling and sequencing

FAW larvae were collected from Wagou and Gando villages in Benin (2017), from Texcoco in Mexico (2009), from French Guiana (1992), and from Petit-Bourg and Port-Louis in Guadeloupe (2013). We obtained gDNA from India, which was used by Sharanabasappa et al.^43^. Genomic DNA was extracted using the Promega Wizard Genomic DNA Kit or the Qiagen DNeasy®lood and Tissue Kit. Libraries for whole genome resequencing were constructed from 1.0 μg of DNA per sample using the NEBNext DNA Library Prep Kit. We performed whole genome sequencing from FAW samples collected in Benin (39 individuals), India (14), Mexico (26), Florida (24), French Guiana (3), and Guadeloupe (4) using the NovaSeq 6000 System with 20X coverage on average with 150 bp paired-end and 300 bp insert length. Then, we combined resequencing data from Santa Isabel in Puerto Rico (15) (NCBI SRA: PRJNA577869), Stoneville in Mississippi (17) (PRJNA494340), and from Citra and Jacksonville in Florida (USA) (24) (PRJNA639296), which were generated for our previous studies^45,47,48^. The resequencing data from Kunming in China (2)^44^ was also included in the analysis. Finally, we took advantage of using resequencing data from the State of Goiás in Brazil (10), from nine districts including Blantyre, Chiradzulu, Machinga, Mulanje, Thyolo, Zomba, Salima, and Karonga, Mzimba, and Nkhata Bay in Malawi (16), and from eight districts including Amolatar, Katakwi, Kumi, Ngora, Pallisa, Soroti, Mbarara and Wakiso in Uganda (7), which were generated by Commonwealth Scientific and Industrial Research Organisation for other studies^49,50^. The total number of geographic locations is 12, which covers a large proportion of the FAW distribution area. The total number of individuals used in this study is 177 (99 from native populations and 78 from invasive populations) (Fig. 1 and Table 1). Since the geographic locations include both North and South America (native populations), West and East Africa, and South and East Asia (invasive populations), the resequencing data represents a large proportion of distribution in FAW.

### Variant calling

Nucleotides with a Phred score less than 20 and adapter sequences were removed from the reads using AdapterRemoval v2.1.7^72^. Reads were mapped against the ver7 reference genome (https://bipaa.genouest.org/sp/spodoptera_frugiperda_pub/download)^48^, which had chromosome-sized scaffolds, using bowtie2 v2.3.4.1 with the -very-sensitive-local preset^73^. The read depth of mapping of resequencing data was 17.72X in the median. Then, we performed local re-assembly of haplotypes using GATK v4.1.2.0^74^. The resulting gvcf files were merged into one gvcf file with CombineGVCFs command and variant calling was performed with GenotypeGVCF command in the same software. Filtering was performed from the resulting VCF (variant calling format) file if QD is lower than 2.0, FS is higher than 60.0, MQ is lower than 40.0, or MQRankSum is lower than − 12.5, or ReadPosRankSum is lower than − 8.0. The total number of genetic variance is 158,277,530, which includes 89,387,997 SNVs (single nucleotide variations). After filtering, 27,117,672 SNVs remained.

### Strain identification

FAW strains were identified using a phylogenetic tree reconstructed from the mitochondrial COX1 gene, which is the universal barcode gene and is also commonly used for FAW strain identification^75^. We first mapped the Illumina reads against mitochondrial genomes (NCBI: KM362176) using bowtie2 v2.3.4.1 with -very-sensitive-local preset^73^, followed by extracting mitochondrial reads using samtools v1.9^76^. Mitochondrial genomes were assembled from these reads, and mitochondrial genes were annotated using MitoZ using the default options^77^. Mitochondrial COX1 sequences were aligned together with a COX1 sequence from a specimen of another *Spodoptera* species, *S. exigua* (Hübner) (NCBI: JX316220), using MUSCLE v3.8.31^78^, and a maximum likelihood phylogenetic tree was reconstructed using MEGA X^79^. We used the Kimura 2-parameter model, and a uniform substitution rate across the alignment was assumed to avoid potential statistical artifacts caused by parameter richness. Non-parametric bootstrapping was performed with 1,000 replications to calculate statistical support at each node. The phylogenetic tree was visualized using iTOL v6^52^. We determined the mitochondrial strains from sfC and sfR clades with the individuals from Mississippi and Puerto Rico, in which strains were already determined for each individual in our previous studies^45,47^.

FAW strains were also identified using the TPI marker. First, we extracted the TPI locus from the vcf file using tabix v1.10.2-3^80^. Principal component analysis was performed from the TPI locus using plink v1.9^81^. Then, the strain of each sample was assigned according to the grouping.

### The inference of origin of the introduction

We identified a native population that was genetically most closely related to the invasive populations from the population structure. The principal component analysis was performed to identify the main groups in FAW using plink v1.9^81^. Ten principal components were extracted, and the main groups in FAW were identified from two major principal components. Significant genetic differentiation between groups was tested using F_ST_^82^ calculated from VCFtools v0.1.15^83^. F_ST_ was calculated from 100 kb windows. The genomic average F_ST_ was calculated from Weir and Cockerham’s weighted mean F_ST_. Significant genetic differentiation between two groups was tested with a permutation test^84^. More specifically, (i) F_ST_ was calculated from two groups, (ii) F_ST_ was calculated from randomly generated groups with 100 replications, and (iii) the proportion of random groups that have higher F_ST_ than real groups was calculated. Since this proportion represents type I statistical error, the proportion was interpreted as a p-value. Ancestry coefficient analysis was performed using admixture v1.3.0 in a wide range of K values^51^.

Phylogenetic relationships with gene flow among the main groups of native populations were estimated to infer evolutionary history using TreeMIX v1.13^85^. First, a new VCF file with individuals from native populations was generated by subsetting the original VCF file with 177 individuals. Second, *S. litura* (Fabricius), which is one of the most closely related species to FAW in the *Spodoptera* genus^86^, was used as an outgroup, and a VCF file was generated by mapping *S. litura* Illumina reads (NCBI SRA, SRX2446353) against the FAW reference genome using bowtie2 with -very-sensitive-local preset^73^ and by variant calling using GATK v4.1.2.0^74^. Third, these two VCF files were merged using VCFtools v0.1.15^83^, while missing data of *S. litura* was not allowed. The number of used SNVs is 697,498. Fourth, the newly merged VCF file was converted to TreeMIX format using vcf2treemix.sh (https://github.com/speciationgenomics/scripts/blob/master/vcf2treemix.sh). Fifth, we ran a TreeMIX binary with the number of migration edges ranging from three to six while the resulting tree was rooted by *S. litura*. The number of SNVs per block was 500 to estimate covariance matrices. Finally, the resulting file was visualized using the internal R library (plotting_funcs.R) in the TreeMix software.

We also used the VCFphylo approach^47^ to infer phylogenetic relationships at the level of individuals out of whole genome sequences. First, the euclidean distance of allele frequency between each pair of individuals was calculated from the biallelic variant positions in which genotypes are determined from all individuals using VCFphylo^47^. Transversional variants were weighted to two. Then, bootstrapping distance matrices were generated with 1,000 replications, and we generated BIO-NJ trees for each matrix using FastME v2.1.5^87^. Then, a consensus tree was made using consense in the Phylip package v3.697^88^. The tree was visualized using iTOL v6^52^.

### The inference of loci under selective sweeps

Potential targets of selective sweeps were identified from the composite likelihood of being targeted by selected sweeps from the site frequency spectrum using SweeD v3.2.1^54^ from the largest 29 scaffolds, which are believed to be chromosome-sized. The proportion of the assembly covered by these 29 scaffolds is 93.52%. The number of grids in which composite likelihood was calculated was 1,000 per scaffold. Based on the assumption that most genomic sequences were not targeted by selective sweeps, outliers of composite likelihood were identified as loci targeted by selective sweeps. The annotation of Cytochrome P450 genes was manually curated, as described in Gouin et al.^46^. The name of the CYP gene was validated by the P450 nomenclature committee^89^.

## Supplementary Information

Supplementary Information.

## Data Availability

The resequencing dataset generated during the current study is available in the NCBI SRA (Accession Number: PRJNA639295).

## Code availability

Computer programming scripts used in this study are available on GitHub (https://github.com/kiwoong-nam/FAW_invasion).
