# Supplementary material for: The evolutionary process of invasion in the fall armyworm (Spodoptera frugiperda)
Source: Sci Rep. 2022 Dec 6;12:21063. doi: 10.1038/s41598-022-25529-z (PMC9727104; doi:10.1038/s41598-022-25529-z)
Supplement: Supplementary file 1 — Supplementary Information. [file 41598_2022_25529_MOESM1_ESM.docx]

Table S1. The list of genes on the potential targets of invasive specific selective sweeps.

| Chromosome | start | end | ID | gene name |
| --- | --- | --- | --- | --- |
| 8 | 8,554,974 | 8,560,903 | SFRUCORN0000021653 | Protein of unknown function |
| 8 | 8,590,850 | 8,597,741 | SFRUCORN0000021660 | Protein of unknown function |
| 8 | 8,563,080 | 8,563,500 | SFRUCORN0000021654 | Protein of unknown function |
| 8 | 8,588,488 | 8,588,871 | SFRUCORN0000021658 | Protein of unknown function |
| 8 | 8,571,015 | 8,595,815 | SFRUCORN0000021655 | Atrial natriuretic peptide receptor 1 |
| 14 | 12,275,316 | 12,282,581 | SFRUCORN0000025984 | Cytochrome p450 CYP9A75 |
| 14 | 12,386,949 | 12,388,669 | SFRUCORN0000025990 | Tubulin beta-3 chain |
| 14 | 12,389,894 | 12,391,590 | SFRUCORN0000025991 | Tubulin beta-3 chain |
| 14 | 12,395,694 | 12,402,075 | SFRUCORN0000025993 | alpha-1,2-mannosyltransferase ALG9 |
| 14 | 12,405,090 | 12,407,146 | SFRUCORN0000025995 | Pre-rRNA-processing protein TSR2 |
| 14 | 12,391,848 | 12,393,676 | SFRUCORN0000025992 | Tubulin beta-3 chain |
| 14 | 12,320,781 | 12,331,540 | SFRUCORN0000025986 | Protein of unknown function |
| 14 | 12,371,977 | 12,379,028 | SFRUCORN0000025988 | Protein of unknown function |
| 14 | 12,404,482 | 12,405,037 | SFRUCORN0000025994 | Protein of unknown function |
| 14 | 12,407,580 | 12,429,248 | SFRUCORN0000025996 | DNA topoisomerase 2-binding protein 1 |
| 29 | 6,570,648 | 6,570,917 | SFRUCORN0000001059 | Probable Transposon |
| 29 | 6,646,496 | 6,651,061 | SFRUCORN0000001051 | Protein of unknown function |
| 29 | 6,596,394 | 6,616,494 | SFRUCORN0000001055 | peptide transporter |
| 29 | 6,654,052 | 6,658,901 | SFRUCORN0000001050 | Protein of unknown function |
| 29 | 6,582,434 | 6,589,323 | SFRUCORN0000001057 | Fer 2 homolog |
| 29 | 6,644,505 | 6,645,033 | SFRUCORN0000001052 | Protein of unknown function |
| 29 | 6,598,209 | 6,599,921 | SFRUCORN0000001056 | carboxylesterase 022a (cxe022a) |
| 29 | 6,573,145 | 6,577,083 | SFRUCORN0000001058 | palmytoyltransferase |
| 29 | 6,693,319 | 6,704,962 | SFRUCORN0000001044 | centrosomal protein |
| 29 | 6,660,376 | 6,665,474 | SFRUCORN0000001048 | odorant receptor 13 |
| 29 | 6,692,263 | 6,692,466 | SFRUCORN0000001045 | Protein of unknown function |
| 29 | 6,671,207 | 6,673,259 | SFRUCORN0000001047 | SPARC-related modular calcium-binding protein |
| 29 | 6,683,531 | 6,691,698 | SFRUCORN0000001046 | SPARC-related modular calcium-binding protein |
| 29 | 6,710,652 | 6,729,471 | SFRUCORN0000001043 | ubiquitin carboxyl-terminal hydrolase |
| 29 | 6,654,220 | 6,659,767 | SFRUCORN0000001049 | Kunitz-type serine protease inhibitor |
| 29 | 6,530,682 | 6,542,329 | SFRUCORN0000001062 | Clock (CLK) |
| 29 | 6,548,052 | 6,549,563 | SFRUCORN0000001061 | Protein of unknown function |
| 29 | 6,554,517 | 6,567,412 | SFRUCORN0000001060 | Uncharacterized, RING domain protein family |
| 29 | 6,513,073 | 6,526,354 | SFRUCORN0000001063 | ABC transporter; multidrug resistance protein homolog 49-like |
| 29 | 20,629,938 | 20,637,386 | SFRUCORN0000010362 | zinc finger and BTB domain-containing protein |
| 29 | 20,638,804 | 20,639,796 | SFRUCORN0000010363 | UDP sugar transporter, probable UST74c |


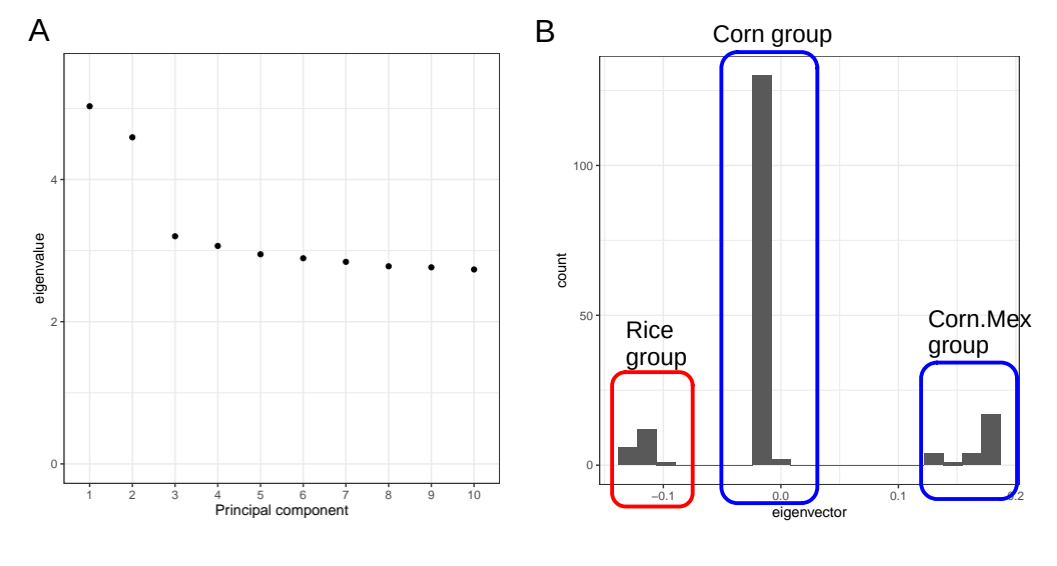
Figure S1. Eigenvalues (A) and eigenvectors (B) of the first component in Fig. 2B. The eigenvectors exhibit three groups (Rice group, Corn group, and Corn.Mex group).

Figure S2. A phylogenetic tree among Corn.Mex, Corn, and Rice groups, together with *Spodoptera litura* as an outgroup with different numbers
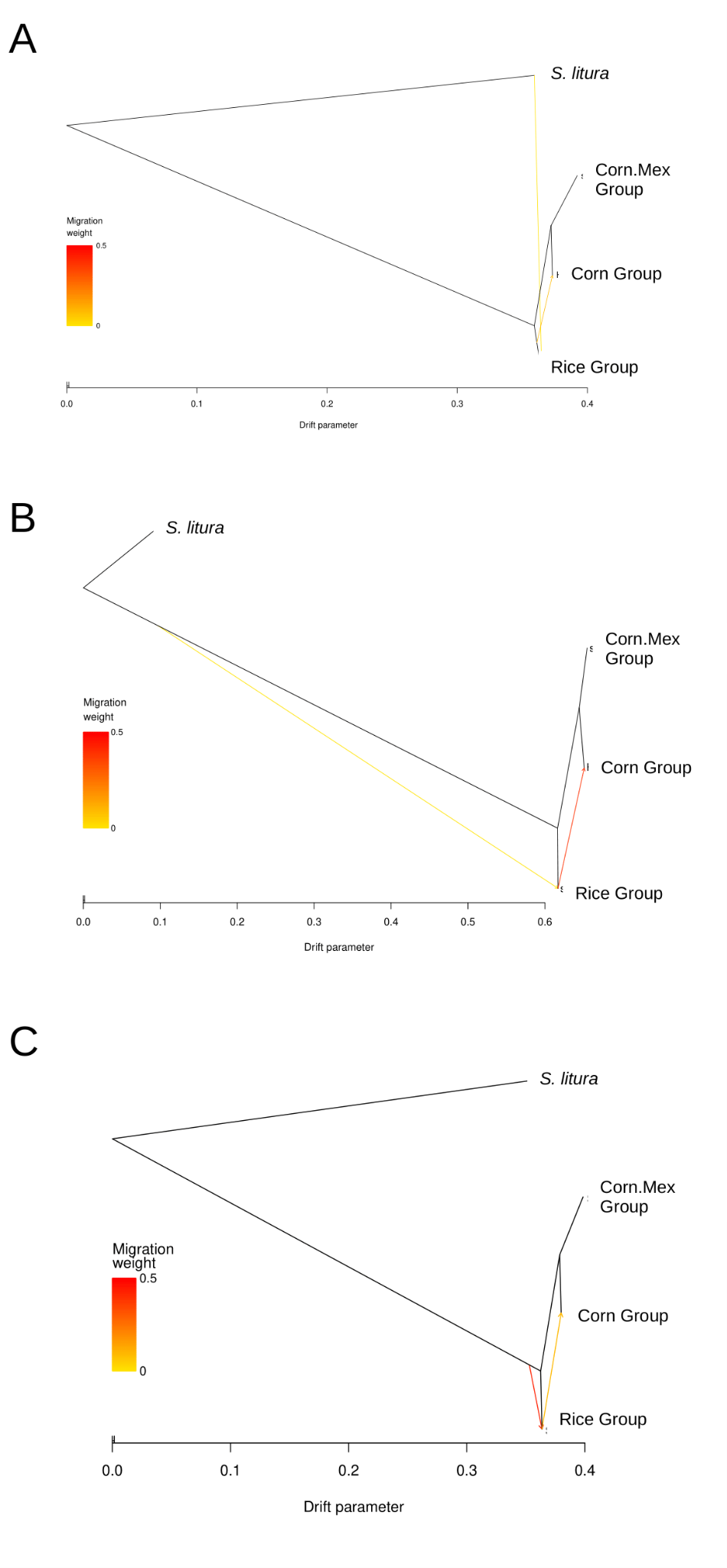
 of migration edges equal to (A) 3, (B) 4, and (C) 5.


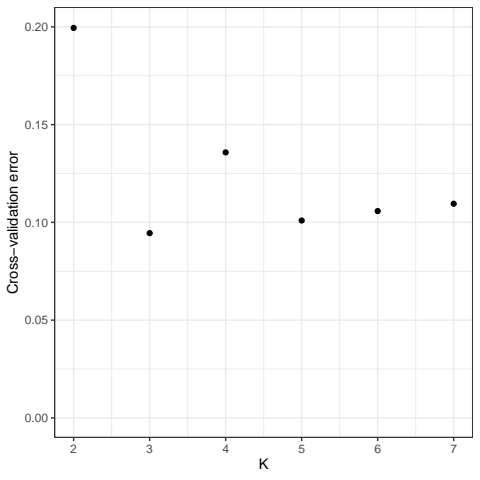


Figure S3. Cross-validation errors for each K value shown in Fig. 3A. The lowest cross-validation error was observed when K = 3, which showed a separation among invasive populations, non-Mexican populations, and the Mexican population.


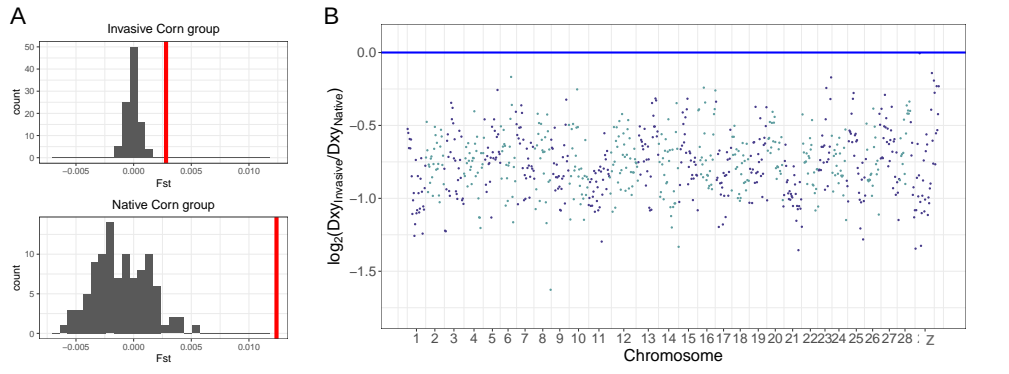
Fig. S4. **Genetic admixture between mitochondrial strains** A. F_ST_ was calculated from the nuclear genomes between the mitochondrial strains (the red vertical bars) or between random groups (histograms) in invasive (the upper panel) or native (the lower panel) individuals of the Corn group. No random groups generated higher F_ST_ than the mitochondrial strains, implying statistically significant nuclear genomic differentiation between the mitochondrial strains (p < 0.01). B. The log-transformed ratio of D_XY_ between the mitochondrial strains in invasive to native populations in the Corn group across the nuclear genomes. If a log-transformed ratio is lower than zero (the blue horizontal bar), invasive populations have lower D_XY_ than native populations.


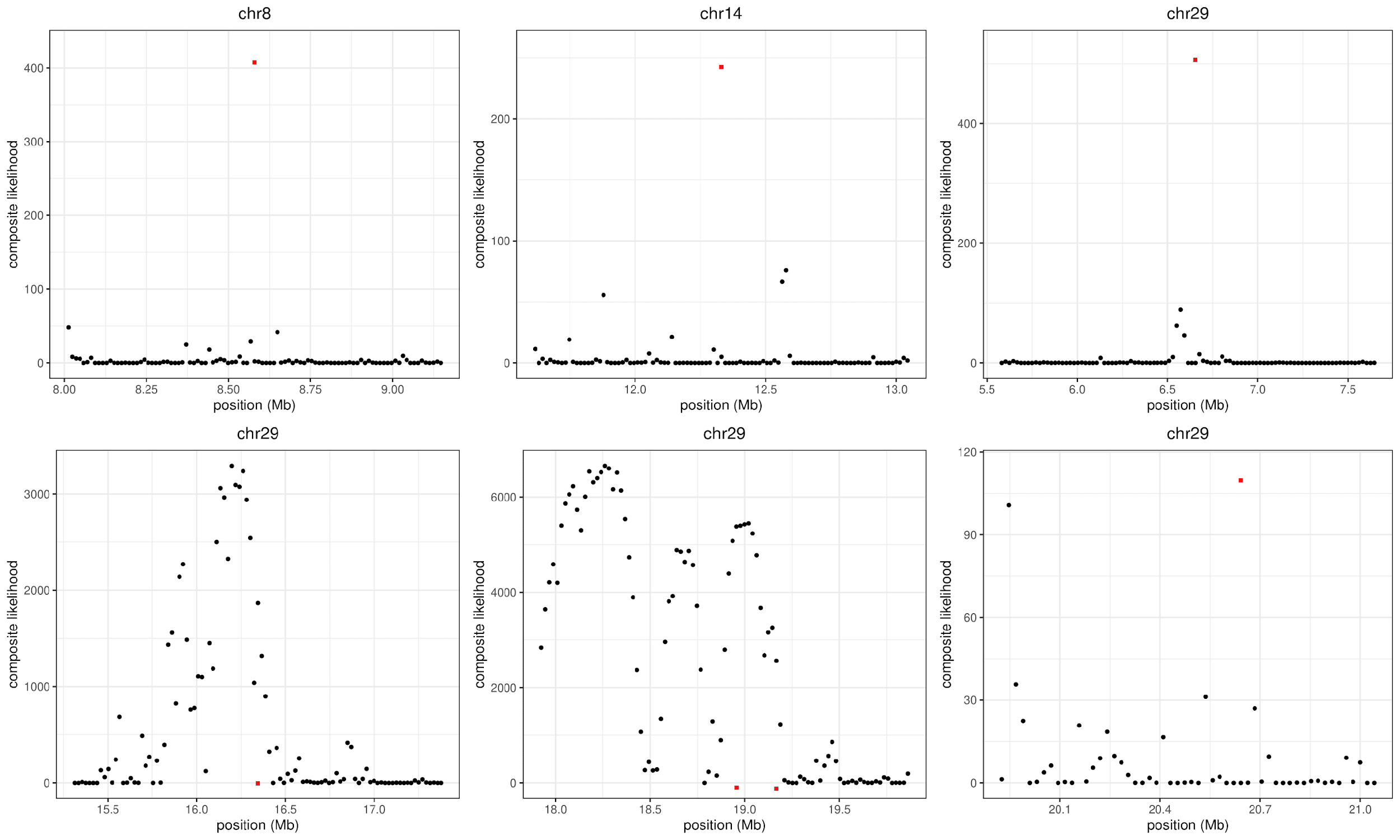


Figure S5. The distribution of composite likelihoods calculated from the native Corn group at the identified seven outliers identified from the invasive populations. The red dots indicate the composite likelihood of invasive populations in the middle of outliers. The native Corn group does not have increased composite likelihoods at the first three and the last outliers. Therefore, we considered that these four outlier loci are potential targets of selective sweeps specific to the invasive populations.
